# Cardiovascular benefits of air purifier in patients with stable coronary artery disease: A randomized single-blind crossover study

**DOI:** 10.3389/fpubh.2022.1082327

**Published:** 2023-01-09

**Authors:** Zhe Liu, Qin Wang, Na Li, Chunyu Xu, Yunpu Li, Jun Zhou, Liu Liu, Haijing Zhang, Yang Mo, Feng Han, Dongqun Xu

**Affiliations:** ^1^China CDC Key Laboratory of Environment and Population Health, National Institute of Environmental Health, Chinese Center for Disease Control and Prevention, Beijing, China; ^2^Chaoyang District Center for Disease Control and Prevention, Beijing, China; ^3^National Institute for Occupational Health and Poison Control, Chinese Center for Disease Control and Prevention, Beijing, China

**Keywords:** air purifier, intervention trial, PM_2.5_, SCAD, drug use, health protection

## Abstract

**Background:**

Exposure to PM_2.5_ will accelerate the progression of cardiovascular diseases. Air purifier can reduce the PM_2.5_ exposure and theoretically alleviate the influence of PM_2.5_ on patients with stable coronary artery disease (SCAD). However, few studies of the protective effect showed significant results because the interferent effects of routine medication had not been taken into account. In order to explore the actual effect on patients with SCAD, we conducted a randomized single-blind crossover air purifier intervention trial.

**Method:**

Levels of PM_2.5_ exposure during intervention and cardiovascular indicators (inflammation, coagulation, plaque stability, and blood lipids) after intervention were detected, meanwhile the information of drug use was obtained by questionnaire. The kinds of drug used by more than 20% of the subjects were sorted out. And the influence of these drugs on cardiovascular indicators was summarized through literature review. Based on that, the drug use was included as a variable in linear mixed effects models that used to analyze the associations between PM_2.5_ exposure reduction by air purifier and cardiovascular indicators.

**Results:**

The result revealed that the interpretation contribution rate of drug use was more than that of PM_2.5_ exposure. The level of C-reactive protein significantly decreased by 20.93% (95%CI: 6.56%, 33.10%), 23.44% (95%CI: 2.77%, 39.39%) and 24.11% (95%CI: 4.21%, 39.69%) on lag1, lag01 and lag02 respectively, while the level of high-density lipoprotein cholesterol significantly increased by 5.10% (95%CI: 0.69%, 9.05%), 3.71% (95%CI: 0.92%, 6.60%) and 6.48% (95%CI: 2.58%, 10.24%) respectively on lag0, lag1 and lag01 associated with an interquartile range decrease of 22.51 μg/m^3^ in PM_2.5_ exposure.

**Conclusion:**

The study shows positive effects of air purifier on SCAD, and also provides methodological reference for future related research.

## 1. Introduction

PM_2.5_ exposure has been confirmed to cause changes of cardiovascular indicators, thus promoting the occurrence and development of cardiovascular diseases (1). Air purifier with high efficiency particulate air filter (HEPA) can significantly reduce the indoor PM_2.5_ concentration (2, 3) then the personal exposure (4). Therefore, there is theoretical basis for using air purifier to reduce health hazards, in areas and seasons with high incidence of PM_2.5_ pollution.

The results of a series of intervention studies demonstrated that the use of air purifiers has different effects on the cardiovascular indicators of different groups. For healthy adults, the use of air purifiers has significant effects on the levels of monocyte chemokines, interleukin-1beta, soluble cluster of differentiation 40 ligand (sCD40L) and endothelin-1 (ET-1) in their circulatory system (5, 6), while the effects on C-reactive protein (CRP) (5, 7–9) and fibrinogen (FIB) (5, 10) are still controversial, and no significant effects on tumor necrosis factor-alpha (TNF-α), interleukin-6 (IL-6) and cluster of differentiation 62 platelet (CD62P) have been found (5, 7, 8, 11). For the elderly, most studies showed no significant impacts on circulatory CRP, TNF-α, IL-6 and FIB levels by using air purifier, except on a physiological index microvascular function (2, 4, 12). But stratification analysis in one correlational study (2) found that the protective effect of air purifiers on microvascular function was not statistically significant in the elderly taking cardiovascular disease medicine. Thus, it can be seen that the conventional cardiovascular disease medicine may interfere the cardiovascular benefits of air purifiers for the elderly.

Patients with stable coronary artery disease (SCAD) usually receive comprehensive drug treatment, including mutiple drugs, to control the development of disease by regulating various functions of the circulatory system. For example, statins are mainly used to regulate the blood lipid balance; β-blockers, angiotensin-converting enzyme inhibitors (ACEI), angiotensin-receptor blockers (ARB) and calcium-channel blockers (Ca-channel blockers) are used to regulate the cardiac load and vasoconstriction; and aspirin is used to inhibit the platelet aggregation. These drugs may independently or synthetically change some cardiovascular indicators, involving inflammation, coagulation, plaque stability and blood lipids, which can also be affected by PM_2.5_ exposure (1, 13, 14). Therefore, it is essential to comprehensively control the interference effects of these drugs, when intervention studies on the protective effect of air purifiers on the cardiovascular system are carried out.

To explore the actual protective effect of PM_2.5_ exposure reduction by air purifiers, a randomized crossover intervention design was adopted to the enrolled patients with SCAD. Air purifiers were used in their residences to reduce the indoor PM_2.5_ concentration. At the same time of PM_2.5_ exposure monitoring, the drug use of the subjects during the intervention was investigated. And levels of the cardiovascular indicators of inflammation, coagulation, plaque stability and blood lipids after the intervention were detected. Interference effects of drug use were controlled in liner mixed effect models, which used to analyze the associations between PM_2.5_ exposure and cardiovascular indicators.

## 2. Materials and methods

### 2.1. Study design and subject recruitment

**Figure 1:**
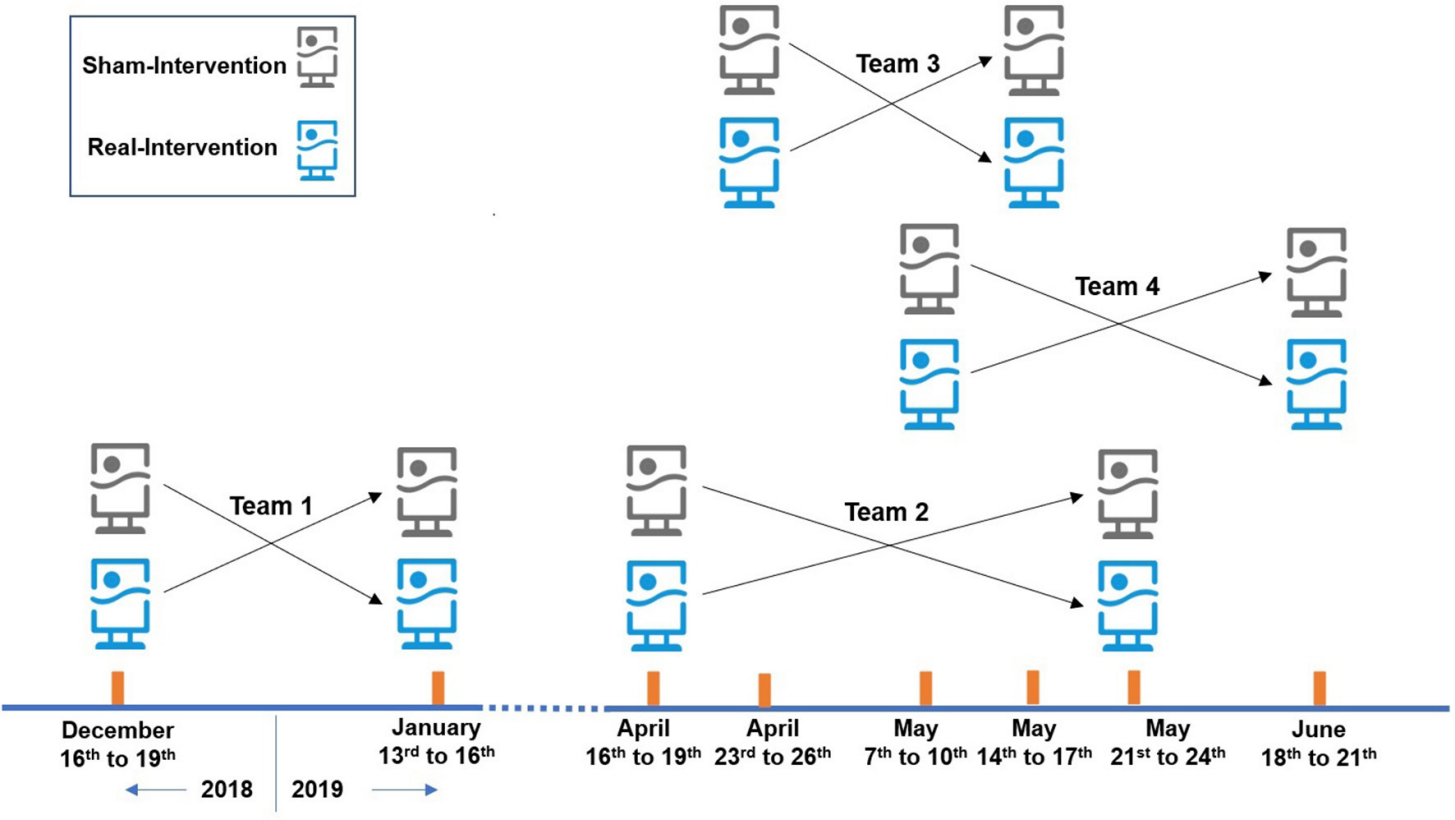
Arrangement of the intervention.

In this study, the randomized single-blind crossover intervention design was adopted. 24 SCAD patients were recruited, and the intervention trial was conducted in their residences, with air purifiers placed in their main activity space. Air purifiers equipped with HEPA filter were “real-intervention,” with a clean air delivery rate of 500 m^3^/h for PM_2.5_, while those without HEPA were “sham-intervention.” The experiment was completed in 4 batches with 6 subjects in each batch. Every intervention period was 3 days and the washout period was more than 14 days. The arrangement of the intervention is shown in Figure 1.

The subjects were recruited from 3 community-level (or above) general hospitals in Beijing, who were clinically diagnosed with SCAD according to the diagnostic requirements of *2013 European Society Cardiology Guidelines on the Management of Stable Coronary Artery Disease* (15) published by the European College of Cardiology and *2010 Chinese Consensus on the Management of Chronic Stable Coronary Artery Disease* (16) issued by China. In addition, the inclusion criteria include: (a) the age was between 55 and 80 years old; and (b) they lived near the hospital for a long time and the house was not decorated within 1 year. The exclusion criteria are as follows: (a) someone in their house smoked or themselves had just quitted smoking for <1 year; (b) patients were diagnosed with acute myocardial infarction within 3 months, or receive percutaneous coronary intervention and bypass transplantation within 6 months; (c) patients wore a pacemaker; and (d) patients suffered from liver injury, cancer and other serious diseases.

During the intervention, the individual exposure to PM_2.5_ and the concentration of PM_2.5_ indoor and outdoor the residence was monitored continuously. The age and body mass index (BMI) of the subjects was investigated, and their travel behaviors, window opening time and medication were recorded. The subjects stayed at home for more than 20 h/d, and the window opened for no more than 2 h/d during the intervention. Drug use records focused on conventional medicine for CAD treatment. Blood samples were collected at the end of each intervention, and the following indicators were detected: (a) inflammation indicators such as IL-6, TNF-α, CRP and FIB; (b) coagulation indicators such as CD62P, CD40L, intercellular adhesion molecule-1 (ICAM-1), nitric oxide (NO) and ET-1; (c) plaque stability indicators such as matrix metalloproteinase-2 (MMP-2) and matrix metalloproteinase-2 (MMP-9); (d) blood lipid indicators such as total cholesterol (CHO), triglyceride (TG), high-density lipoprotein (HDL-C) and low-density lipoprotein (LDL-C).

### 2.2. PM_2.5_ exposure

Particulate matter sampling pumps MicroPEM^TM^ (RTI Company, USA) based on the principle of light scattering were used to monitor PM_2.5_ concentration with sampling flow of 500 mL/min. It can monitor the environmental temperature and relative humidity (RH) simultaneously. Daily average concentration of PM_2.5_ was calculated from 8 a.m. to 8 a.m. Lag and moving average levels of PM_2.5_ exposure are represented by “lag.”

Sampling pumps for monitoring the indoor PM_2.5_ concentration were placed in the open area of the house to avoid air conditioner vent, kitchen, doors and windows, about 1.5 m above the ground, more than 1 m far from the wall, and at least 2 m far from the air purifier. Sampling pumps for monitoring the outdoor PM_2.5_ concentration were placed outside the residential window and avoid air conditioner unit and kitchen and its air outlet. Sampling pumps for monitoring individual exposure to PM_2.5_ were taken along by the subjects.

### 2.3. Blood collection and indicator measurements

Fasting blood samples (5 mL anticoagulant and 5 mL non-anticoagulant) were collected from 8:00 to 9: 00 on the day at the end of each intervention. IL-6, TNF-α, CRP, FIB, CD62P, CD40L, ICAM-1, NO, ET-1, MMP-2 and MMP-9 levels were tested by a professional testing institution. CHO, TG, HDL-C and LDL-C levels were tested by the laboratory of the hospital. IL-6, TNF-α, CD62P, CD40L and ICAM-1 was detected by a bead-based multiple flow cytometry on Luminex 200 system (Luminex Corporation, Austin, TX, USA) with chips (IL-6 and TNF-α: Merck Millipore, Germany; CD62P, CD40L and ICAM-1: R&D, USA). CRP, NO, MMP-2, MMP-9, FIB and ET-1 was separately detected by ELISA kit (CRP, NO, MMP-2, MMP-9: R&D, USA; FIB: NOVUS, USA; ET-1: Elabscience Biotechnology Co. Ltd, Wuhan, China).

In addition, a compound index-atherogenic index of plasma (AIP) was calculated by the results of TG and HDL-C levels and the formula: $\text{AIP}=\text{log}\left(\;\frac{\text{TG}}{\text{HDL}-\text{C}}\;\right)$.

### 2.4. Statistical analyses

Descriptive statistics were conducted for general characteristics of the environmental exposure and effects. Data with normal distribution were described by mean and standard deviation (Mean±SD), and data with skewed distribution were described by median and interquartile range as M (P_25_, P_75_). The paired Student's *t*-test was employed to compare the PM_2.5_ concentrations between two intervention periods, or the indoor and outdoor environment. The Linear mixed effect models were conducted to estimate the correlation between PM_2.5_ exposure and cardiovascular indicators. The results were reported by the percentage change of indicators associated with an interquartile range (IQR) decreases of PM_2.5_ exposure. The interpretation contribution rate of independent variables in the model were calculated by the determination coefficient R^2^ of independent variables/ of the model.

In the linear mixed effect models, the “effective drug” variable is defined as one or more kinds of the effective drug had been taken by the subject (dichotomous variable). The kinds of drug need to be controlled were selected according to *Guidelines for Rational Drug Use of Coronary Artery Disease (2nd Edition)* (17) and the actual medication of the subjects (taken by more than 20% of the subjects). Whether the drug is effective was determined by searching the literatures on “Pubmed,” “Web of Science,” “China National Knowledge Infrastructure,” “Wanfang” and “VIP” (before December 30, 2021). It needs to meet the following requirements: (a) there were two or more case-control trials or pre-and post-treatment control trials of cardiovascular disease patients treated with the drug, and the results of the trials were statistically significant; or (b) there was a case-control trial or pre-and post-treatment control trial of cardiovascular disease patients treated with the drug, and the results were statistically significant, and there was one or more animal experiment of the drug, and the results were statistically significant. The Newcastle-Ottawa Scale (NOS) was used to assess the quality of literatures relevant to case control/pre-and post-control studies in patients with cardiovascular disease, and the CAMARADES Scales was used to assess the quality of literatures relevant to animal studies. Literatures with a score more than 6 points would be included in the summary.

Influencing factors of PM_2.5_ exposure and cardiovascular indicators need to be considered as independent variables in the linear mixed effects models. It includes the “effective drug,” age, gender and BMI, as well as temperature and relative humidity. The stepwise elimination method based on the principle of minimization of Akaike's Information Criterion (AIC) was adopted for the validity of models. We screened three additional independent variables on the basis of preserving key variables (PM_2.5_ exposure and the “effective drug”). The model was established for each indicator respectively, given the influencing factors differences.

The parameters of the model were tested by bilateral *t*-test, with statistical significance of 0.05. The “lme4” package and “r2glmm” package in the R (Version 4.0.3) was separately used for linear mixed effect model and R^2^ calculation.

### 2.5. Quality control

The monitoring instruments were cleaned and calibrated before sampling. Instrument condition review was done at the end of the first and last day of each intervention. The blood samples were collected by the nurses. Blood lipid indicators were detected on the same day, and samples were stored at −80°C before further detection. The detection process was controlled strictly in accordance with the testing procedures and the requirements of the chips/kits.

The subjects of the study were trained by professional personnel before recording the questionnaire themselves. Questionnaires were re-checked at the end of the first and last day of each intervention for ensuring recording accuracy. Questionnaire and data input were done by two personnel, and then reviewed and analyzed by special personnel.

## 3. Results

### 3.1. The general characteristics of the subjects

The residences of the subjects were all located within 5 km of the hospitals, in apartment buildings built from 2003 to 2015, with an area between 45.1 and 96.0 m^3^. The windows of the rooms were made of plastic steel or aluminum alloy to ensure the sufficient air tightness. The average age of the subjects was 65.3 ± 5.2 years old, and the ratio of male to female was 14:10. The average BMI of the subjects was 26.0 ±2.4 kg/m^2^. The drug use during the intervention is shown in Table 1.

**Table 1:** The general characteristics of the participations.

**Items**	**Categories**	**Result**
Age (Mean±SD)		65.3 ± 5.2 years
Gender (n)	Male	14
	Female	10
BMI (Mean±SD)		26.0 ± 2.4 kg/m^2^
Drug Use (n)		
	Aspirin	18
	Statins	18
	ACEI/ARB	14
	Clopidogrel	13
	β-blockers	7
	Ca-channel blockers	5

### 3.2. Air purification effect and individual PM_2.5_ exposure level

The 72 h average indoor PM_2.5_ concentration in the real-intervention period was 40.58% of the outdoor. It was significantly reduced by 40.04% when compared with the sham-intervention period. The change of individual PM_2.5_ exposure level was consistent with indoor concentration. During the real-intervention period, the individual PM_2.5_ exposure level was reduced by 22.72% when compared with the sham-intervention period. The use of air purifiers in residences can effectively reduce the indoor PM_2.5_ concentration, and then the individual PM_2.5_ exposure (Table 2).

**Table 2:** Comparison of indoor, outdoor and individual exposure to PM_2.5_ concentrations.

**Intervention**	**Scene**	**PM_2.5_/μg/m^3^ (Mean±SD)**	**Mean difference with sham/μg/m^3^ (95% CI)**	**Mean difference with outdoor /μg/m^3^ (95% CI)**
Real	Indoor	26.24 ± 11.26	−17.52^*^ (−31.93, −3.09)	−38.44^*^ (−51.78, −25.09)
	Outdoor	64.67 ± 34.45	4.66 (−21.98, 31.31)	—
	Individual	32.65 ± 13.18	−9.60 (−21.84, 2.63)	—
Sham	Indoor	43.76 ± 29.11	—	−16.25^*^ (−23.55, −8.95)
	Outdoor	60.01 ± 36.90	—	—
	Individual	42.25 ± 28.82	—	—

* p < 0.05.

### 3.3. Drug to be controlled and their effects on indicators

According to the questionnaire survey, 6 kinds of drug were taken by more than 20% of the subjects during the intervention period: statins, β-blockers, Ca-channel blockers, clopidogrel, aspirin and ACEI/ARB. Based on literatures review (Supplementary Tables S1–S6) (18–77), the effects of these 6 kinds of drug on the indicators of inflammation, coagulation, plaque stability and blood lipids were determined, as shown in Table 3.

**Table 3:** Effects of 6 kinds of drug on cardiovascular indicators.

	**Statins**	**β-blockers**	**Ca-channel blockers**	**Clopidogrel**	**Aspirin**	**ACEI/ARB**
IL-6	•^a^	•	•		•	•
TNF-α	•	•	•	•	•	•
CRP	•	•	•	•	•	•
FIB	•	•				•
CD62P	•			•	•	
CD40L	•			•		
ICAM-1	•					•
NO					•	
ET-1	•	•				
MMP-2	•		•	•		
MMP-9	•		•	•		•
CHO	•	•			•	
TG	•	•				
HDL-C	•					
LDL-C	•	•			•	
AIP	•	•				

a •Based on literatures review, the effects of drug on the indicators were determined.

### 3.4. Model selection and interpretation contribution rate of variables

In this study, each cardiovascular indicator was selected by minimizing AIC in the linear mixed effect analysis model, except for CHO and LDL-C. Based on the previous association research of blood lipid indicators, the influence of age, gender and BMI variables were greater than that of ambient temperature and RH. And the AIC values of CHO and LDL-C models (“log(**Indicator**) **~PM**_**2.5**_**+Age+Gender+BMI+ED+**(**1**|**ID**)” and “log(**Indicator**) **~PM**_**2.5**_**+Gender+BMI+Temp+ED+** (**1**|**ID**)”) were similar, which were 12.74 vs. 12.21 and 47.03 vs. 46.54, respectively, so “log(**Indicator**) **~PM**_**2.5**_**+Age+Gender+BMI+ED+**(**1**|**ID**)” was selected as the model. The final selection of linear mixed effect models for each cardiovascular indicator is shown in Supplementary Table S7.

The determination coefficient R^2^ of the independent variables of each model and its interpretation contribution rate in the model were calculated (Supplementary Table S7), we found that in the models corresponding to IL-6, TNF-α, FIB, CD62P, ICAM-1, ET-1, MMP-9, CHO, TG and AIP, the contribution rate of “effective drug” to the interpretation of the model was greater than that of PM_2.5_ exposure, and the contribution rate of “effective drug” to the interpretation of IL-6, TG and AIP models was more than 58%. In the model corresponding to the CRP, CD40L, NO, HDL-C and LDL-C, the interpretation contribution rate of PM_2.5_ exposure to the model was slightly greater than that of “effective drug.” In the model corresponding to MMP-2, the interpretation contribution rate of PM_2.5_ exposure to the model was much greater than that of drug intake (60% vs. 3%).

### 3.5. Effects of PM_2.5_ reduction on inflammation indicators

**Figure 2:**
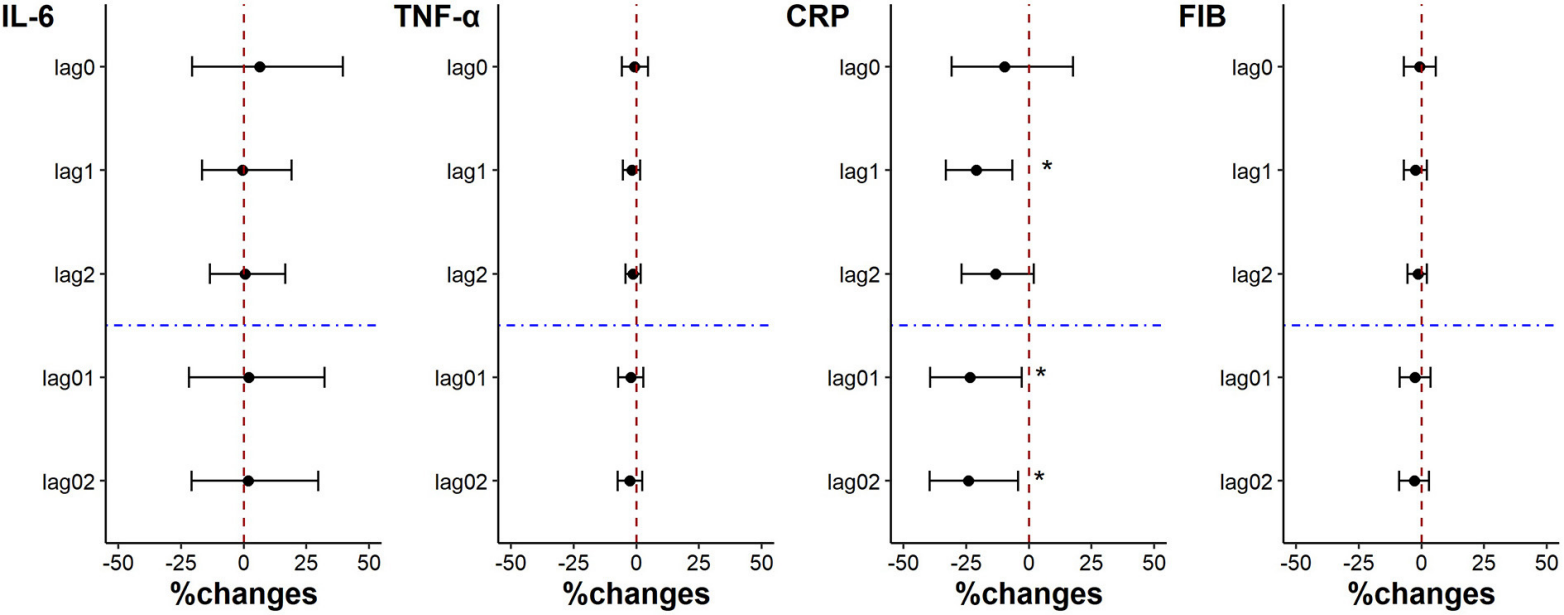
Percent changes in inflammation indicators associated with an IQR decrease in PM_2.5_ exposure. *p < 0.05.

The levels of TNF-α, CRP and FIB were decreased with each IQR decrease (22.51 μg/m^3^) of PM_2.5_ exposure on lag0 when the subjects used the “real” air purifier, but the changes were not statistically significant. And CRP level of the subjects was significantly reduced by 20.93% (95%CI: 6.56%, 33.10%), 23.44% (95%CI: 2.77%, 39.39%) and 24.11% (95%CI: 4.21%, 39.69%) for each PM_2.5_ exposure IQR decrease, on lag1, lag01, and lag02 (Figure 2).

### 3.6. Effects of PM_2.5_ reduction on coagulation indicators

**Figure 3:**
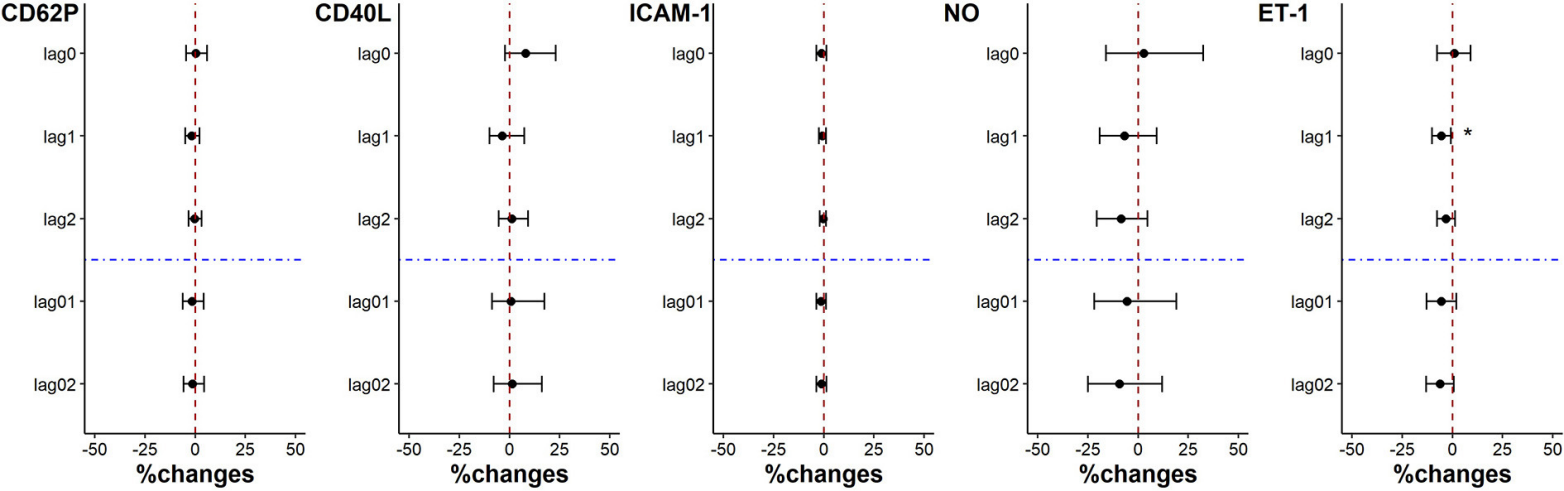
Percent changes in coagulation indicators associated with an IQR decrease in PM_2.5_ exposure. *p < 0.05.

There was no statistically significant change in coagulation indicators when the individual PM_2.5_ exposure was decreased by each IQR on lag0, lag1, lag2, lag01, and lag02. Except that ET-1 level was significantly reduced by 5.64% (95%CI: 0.83%, 10.23%) for each PM_2.5_ exposure IQR decrease on lag1 (Figure 3).

### 3.7. Effects of PM_2.5_ reduction on plaque stability indicators

**Figure 4:**
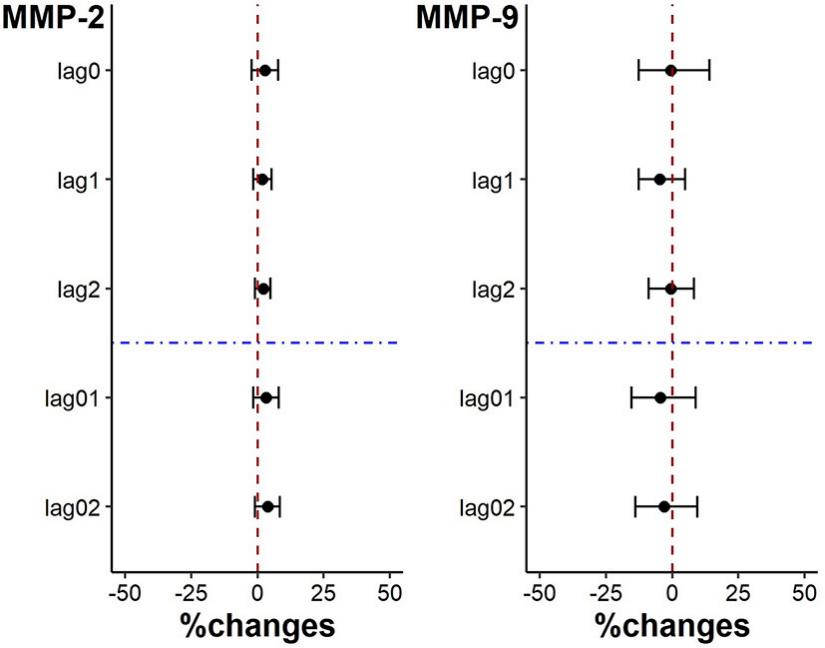
Percent changes in plaque stability indicators associated with an IQR decrease in PM_2.5_ exposure.

There was no significantly change in plaque stability indicators. And while the level of MMP-9 showed a downward trend for each IQR decrease of PM_2.5_ exposure on lag0, lag1, lag2, lag01 and lag02, the level of MMP-2 level showed an opposite trend (*p* > 0.05) (Figure 4).

### 3.8. Effects of PM_2.5_ reduction on blood lipid indicators

**Figure 5:**
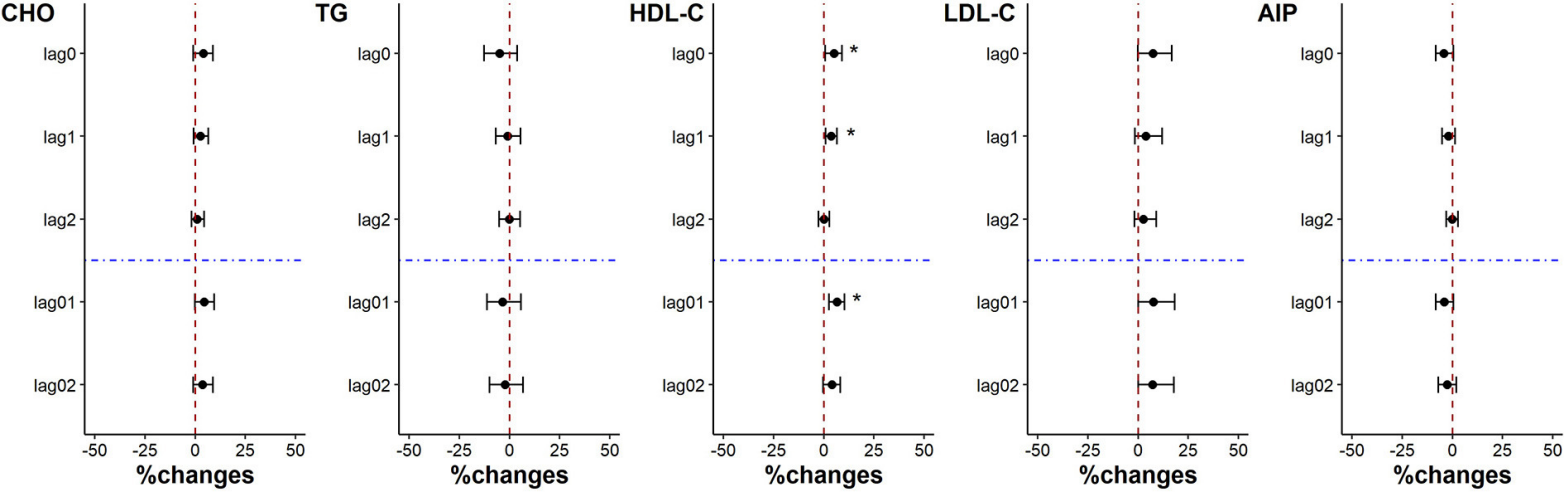
Percent changes in blood lipid indicators associated with an IQR decrease in PM_2.5_ exposure. *p < 0.05.

The levels of CHO, HDL-C and LDL-C increased associated with each IQR decrease of PM_2.5_ exposure on lag0, lag1, lag2, lag01 and lag02. But only the increase of HDL-C was significant on lag0, lag1 and lag01, which was 5.10% (95%CI: 0.69%, 9.05%), 3.71% (95%CI: 0.92%, 6.60%) and 6.48% (95%CI: 2.58%, 10.24%) respectively. Although with no statistical significance, the level of AIP showed a decreasing trend on lag1 and lag01 (*p* = 0.06 and *p* = 0.07, respectively) with the decrease of PM_2.5_ exposure. There was no significant change in the level of TG (Figure 5).

## 4. Discussion

Most of previous air purifiers intervention studies of the elderly did not consider the role of medication, leading to bias in results. In this study, the use of 6 conventional SCAD drugs (statins, β-blockers, Ca-channel blockers, ACEI/ARB, clopidogrel and aspirin) was combined and included into the linear mixed effect model in the form of one “effective drug” variable to analyze the influence of PM_2.5_ exposure on indicators, so as to establish a set of evaluation methods for the health protection effect of air purifier on patients with SCAD. And through a randomized crossover intervention study with small sample size, using the established evaluation method, it was found that the use of “true” air purifier to reduce the PM_2.5_ exposure level of patients with stable CAD had a certain protective effect on the levels of inflammatory indicator CRP and lipid indicator HDL-C under the control of the influence of drugs. But there were no significant effects on other inflammation and lipid indicators, or on coagulation and plaque stability indicators. It is suggested that CRP and HDL-C may be sensitive indicators of the acute effect of PM_2.5_ exposure on SCAD patients, and it is more worthy of attention in the air purifier intervention trials for stable CAD patients. Therefore, the method established in this study provided methodological reference for relevant studies, and the results of the intervention study broadened the understanding on health protection for patients with stable CAD using the air purifier, which has an important public health significance.

Inflammation is recognized as a key step in the development of CAD. Clinical studies have indicated that inflammation indicator CRP is a risk biomarker of atherosclerosis, which can mediate atherosclerosis at different stages (78). Elevated CRP level can predict the occurrence of cardiovascular events (79). In previous intervention studies of air purifiers for healthy adults, a significant association was found between the decrease of CRP and PM_2.5_ exposures levels (8, 9), confirming the health protection of air purifiers. However, no significant association has been found in studies for the elders, including those with cardiovascular disease (7). This study found that CRP level in patients with SCAD significantly decreased by 20.93 to 24.11% when the daily exposure to PM_2.5_ was reduced by 22.51 μg/m^3^ on day lag1, lag01, and lag02. After the interference of drug use was controlled, the air purifiers were found to have a protective effect on CRP levels of the subjects. In addition, the study revealed that the interpretation contribution rate of drug use to the analysis model corresponding to CRP was similar with that of PM_2.5_ exposure, which further illustrates the necessity of controlling the drug use of the subjects in the air purifier intervention studies for patients with SCAD and even patients with cardiovascular diseases who need to take drugs daily.

Blood lipid is one of the risk factors to be controlled in patients with CAD. The meta-analysis (14) showed that for each 10 μg/m^3^ increase in PM_2.5_ long-term exposure, the levels of CHO and LDL-C were increased significantly by 4.53 and 5.36%, respectively, with no significant change in HDL-C and TG levels. In a large cohort study conducted by Wu et al. (80) on middle-aged women (42–52 years old), it was found that the HDL-C level changed significantly by −0.7% when the average yearly concentration of PM_2.5_ was increased by 3 μg/m^3^, but the correlation between the average daily concentration of PM_2.5_ and HDL-C level was not statistically significant. At present, there are only a few population-based studies on the acute effects of PM_2.5_ exposure on blood lipid indicators, and there is no effective evidence to prove a significant correlation between PM_2.5_ exposure and changes in blood lipid indicators, which may be due to the failure to control the effects of drug use on blood lipid. In this study, it's showed that the interpretation contribution rate of drug use to the model of HDL-C was similar to that of PM_2.5_ exposure. And under controlling the drug effects, we analyzed the association between exposure to PM_2.5_ and acute effect of HDL-C in patients with SCAD. The AIP of the subjects in this study tended to improve with the decrease of PM_2.5_ exposure (*p* = 0.06 and 0.07), which proved, together with the results of HDL-C, that air purifiers used to reduce PM_2.5_ exposure might have a positive effect on the improvement of the quality of lipid-related indicators in patients with SCAD. These findings not only illustrate the necessity of considering drug use in relevant studies, but also provide experimental epidemiological evidence for the research of the association between PM_2.5_ exposure and acute effect of blood lipid indicators.

In the environmental health studies of SCAD patients, medications have a greater impact on cardiovascular indicators than environmental factors, so it's important to control their interference with the results. Stratified analyses by single kind of drug usage are usually used for this purpose (2). However, this stratified analysis is too simple considering that SCAD patients often require multiple drugs to control disease progression. The approach used in this study was to integrate multiple medications into an “effective drug” variable, which not only allows for a comprehensive consideration of single and combination drug use, but also reduces the sample size required for such studies.

The intervention study considered the influence of daily drug use on cardiovascular indicators of the patients with SCAD, and revealed the real protective effect of air purifiers on the patients with SCAD after reducing PM_2.5_ exposure level. However, compared with other air purifier intervention trials, the study is an experimental epidemiological study with small sample size, which may have limitation on the research results. The limitation had been noted, and individual measurements had been used to more precisely evaluated PM_2.5_ exposure levels of each study subject, which in turn could increase the accuracy of PM_2.5_ exposure-response evaluation results. In addition, the small sample size limits the stratified analysis for different drug use. If the sample size is expanded, the interference effect of various drug on the results of PM_2.5_ impacts can be analyzed in depth. When considering the influence of drug on cardiovascular indicators, only six kinds of conventional drug for coronary heart disease were controlled in the study. With the development and application of new drug, as well as the new discovery of the effect of drug on cardiovascular indicators, it will be necessary to conduct in-depth studies and to reveal the protective effect of air purifiers on people with cardiovascular diseases by expanding the sample size and increasing the control of the effects of other drug in the future.

## 5. Conclusions

In the evaluation method established in this study, the way to control the influence of drug use can provide methodological reference for future research in related fields, and the comprehensive analysis of the protective effects of air purifiers on cardiovascular system from multiple indicators of inflammation, coagulation, plaque stability and blood lipid can also provide scientific basis for screening sensitive indicators of air purification protection for patients with SCAD. In addition, through a randomized crossover intervention study with small sample size, the study found that the use of air purifier had a clear protective effect on patients with SCAD, in the case of daily routine medication management for disease management, and could significantly improve patients' inflammation indicator CRP and blood lipid indicator HDL-C. It shows that using air purifiers is one of the effective measures to protect the health of patients with SCAD in PM_2.5_ polluted weather.

## Data availability statement

The original contributions presented in the study are included in the article/Supplementary material, further inquiries can be directed to the corresponding author.

## Ethics statement

The study was approved by the Ethics Committee of National Institute of Environmental Health, Chinese Centre for Disease Control and Prevention and all participants provided their written informed consent.

## Author contributions

ZL, QW, NL, CX, YL, and DX: conceptualization and methodology. ZL and NL: project administration. ZL, NL, CX, YL, JZ, LL, HZ, YM, and FH: investigation. ZL and CX: formal analysis. ZL: visualization and writing–original draft. DX: writing–review and editing, supervision, and funding acquisition. All authors have read and agreed to the published version of the manuscript.
