# Supplementary material for: Cardiovascular benefits of air purifier in patients with stable coronary artery disease: A randomized single-blind crossover study
Source: Front Public Health. 2023 Jan 9;10:1082327. doi: 10.3389/fpubh.2022.1082327 (PMC9868303; doi:10.3389/fpubh.2022.1082327)
Supplement: Supplementary file 1 [file Table_1.docx]

Supplementary Material

# Table S1 The literatures on the effect of statins on the cardiovascular indicators

| ID | Drug names | Subjects | Indicators | Change ^a^ | Medication Duration | Sample Size  (n) | Age | References | Design | NOS/  CAMARADES  Score |
| --- | --- | --- | --- | --- | --- | --- | --- | --- | --- | --- |
| 1 | Atorvastatin | Patients with undergoing coronary artery surgery | IL-6 | ↓ | 3W | 29:28 | 61.3±7.7:61.8±7.4 | (18) | case-control | 8 |
|  |  |  |  |  |  |  |  |  |  |  |
| 2 | Atorvastatin | Patients with hyperlipidemia | TNF-α | ↓ | 8W | 45 | 50±2(18-71) | (19) | pre- and post-control | 7 |
|  |  |  | IL-6 | ↓ |  |  |  |  |  |  |
|  |  |  |  |  |  |  |  |  |  |  |
| 3 | Rosuvastatin | Patients with hypertension complicated with dyslipidemia | IL-6 | ↓ | 12W | 16:14 | 56±8.8:54±8.01 | (20) | case-control | 8 |
|  |  |  | TNF-α | ↓ |  |  |  |  |  |  |
|  |  |  | CHO | ↓ |  |  |  |  |  |  |
|  |  |  | LDL-C | ↓ |  |  |  |  |  |  |
|  |  |  | TG | ↓ |  |  |  |  |  |  |
|  |  |  | HDL-C | ↑ |  |  |  |  |  |  |
|  |  |  |  |  |  |  |  |  |  |  |
| 4 | Atorvastatin | Patients with unstable angina | CRP | ↓ | 16W | 2322 | —— | (21) | pre- and post-control | 8 |
|  |  |  |  |  |  |  |  |  |  |  |
| 5 | Atorvastatin | Patients with SCAD & high SCAD risk | CRP | ↓ | 1M/3M | 155 | —— | (22) | pre- and post-control | 8 |
| ID | Drug names | Subjects | Indicators | Change ^a^ | Medication Duration | Sample Size  (n) | Age | References | Design | NOS/  CAMARADES  Score |
| 6 | Atorvastatin | Patients with CAD | CHO | ↓ | 12M | 28 | —— | (23) | pre- and post-control | 9 |
|  |  |  | HDL-C | ↑ |  |  |  |  |  |  |
|  |  |  | LDL-C | ↓ |  |  |  |  |  |  |
|  |  |  | TG | ↓ |  |  |  |  |  |  |
|  |  |  | ICAM-1 | ↓ |  |  |  |  |  |  |
|  |  |  | CD62P | ↓ |  |  |  |  |  |  |
|  |  |  |  |  |  |  |  |  |  |  |
|  | Simvastatin | Patients with CAD | CHO | ↓ | 12M | 30 | —— | (23) | pre- and post-control |  |
|  |  |  | HDL-C | ↑ |  |  |  |  |  |  |
|  |  |  | LDL-C | ↓ |  |  |  |  |  |  |
|  |  |  | TG | ↓ |  |  |  |  |  |  |
|  |  |  | ICAM-1 | ↓ |  |  |  |  |  |  |
|  |  |  | CD62P | ↓ |  |  |  |  |  |  |
|  |  |  |  |  |  |  |  |  |  |  |
| 7 | Simvastatin | Patients with hyperlipidemia | IL-6 | ↓ | 2M | 25:20 | 59±13:55±11 | (24) | case-control | 8 |
|  |  |  | sCD62P | ↓ |  |  |  |  |  |  |
|  |  |  | sCD40L | ↓ |  |  |  |  |  |  |
|  |  |  |  |  |  |  |  |  |  |  |
| 8 | Atorvastatin | Patients with hyperlipidemia | sCD40L | ↓ | 4W | 32 | 60.3±13.2 | (25) | pre- and post-control | 8 |
|  |  |  |  |  |  |  |  |  |  |  |
| 9 | Simvastatin | Patients with SCAD | ET-1 | ↓ | 2W | 16 | —— | (26) | pre- and post-control | 8 |
| ID | Drug names | Subjects | Indicators | Change ^a^ | Medication Duration | Sample Size  (n) | Age | References | Design | NOS/  CAMARADES  Score |
| 10 | Pravastatin | Patients with hypertension complicated with dyslipidemia | ET-1 | ↓ | 16W | 25 | 53±2 | (27) | pre- and post-control | 7 |
|  |  |  |  |  |  |  |  |  |  |  |
| 11 | Atorvastatin | Patients with SCAD | FIB | ↓ | 12W | 36 | 53±9 | (28) | pre- and post-control | 8 |
|  |  |  |  |  |  |  |  |  |  |  |
| 12 | Fluvastatin | Patients with CAD complicated with dyslipidemia | FIB | ↓ | 24W | 82:86 | 57±8:56±8 | (29) | case-control | 8 |
|  |  |  |  |  |  |  |  |  |  |  |
| 13 | Atorvastatin | Patients with hyperlipidemia | hsCRP | ↓ | 12W | 34 | 59.2±9.3 | (30) | pre- and post-control | 8 |
|  |  |  | MMP-9 | ↓ |  |  |  |  |  |  |
|  |  |  |  |  |  |  |  |  |  |  |
|  | Rosuvastatin | Patients with hyperlipidemia | hsCRP | ↓ | 12W | 35 | 57.7±11.1 |  |  |  |
|  |  |  | MMP-9 | ↓ |  |  |  |  |  |  |
|  |  |  |  |  |  |  |  |  |  |  |
| 14 | Simvastatin | Patients with CAD complicated with dyslipidemia | MMP-9 | ↓ | 14W | 13 | 67±8 | (31) | pre- and post-control | 9 |
|  |  |  |  |  |  |  |  |  |  |  |
| 15 | Simvastatin | Patients with CAD complicated with dyslipidemia | CRP | ↓ | 14W | 32:31 | 62±7:62±8 | (32) | case-control | 8 |
|  |  |  | TNF-α | ↓ |  |  |  |  |  |  |
|  |  |  | MMP-9 | ↓ |  |  |  |  |  |  |
| ID | Drug names | Subjects | Indicators | Change ^a^ | Medication Duration | Sample Size  (n) | Age | References | Design | NOS/  CAMARADES  Score |
| 16 | Rosuvastatin | Patients with CHF | MMP-2 | ↓ | 1M | —— | —— | (33) | pre- and post-control | 9 |
|  |  |  | MMP-9 | ↓ |  | —— | —— |  |  |  |
|  |  |  |  |  |  |  |  |  |  |  |
| 17 | Pravastatin | Patients with CAD | MMP-2 | ↓ | 2M | 24 | 68±2 | (34) | pre- and post-control | 8 |
|  |  |  |  |  |  |  |  |  |  |  |
| 18 | Simvastatin | Patients with CAD complicated with dyslipidemia | MMP-9 | ↓ | 8W | 27 | 53 | (35) | pre- and post-control | 9 |
|  |  |  |  |  |  |  |  |  |  |  |
| 19 | Atorvastatin | Patients with hyperlipidemia (64% with CAD) | LDL-C | ↓ | 6W | 639 | 61.7(18-80) | (36) | pre- and post-control | 9 |
|  |  |  | TG | ↓ |  |  |  |  |  |  |
|  |  |  | CHO | ↓ |  |  |  |  |  |  |
|  |  |  | HDL-C | ↑ |  |  |  |  |  |  |
|  |  |  |  |  |  |  |  |  |  |  |
|  |  | Patients with hyperlipidemia (63% with CAD) | LDL-C | ↓ | 6W | 641 | 61.5(18-80) |  |  |  |
|  |  |  | TG | ↓ |  |  |  |  |  |  |
|  |  |  | CHO | ↓ |  |  |  |  |  |  |
|  |  |  | HDL-C | ↑ |  |  |  |  |  |  |
| ID | Drug names | Subjects | Indicators | Change ^a^ | Medication Duration | Sample Size  (n) | Age | References | Design | NOS/  CAMARADES  Score |
| 19 | Simvastatin | Patients with hyperlipidemia (62% with CAD) | LDL-C | ↓ | 6W | 207 | 61.3(18-80) | (36) | pre- and post-control | 9 |
|  |  |  | TG | ↓ |  |  |  |  |  |  |
|  |  |  | CHO | ↓ |  |  |  |  |  |  |
|  |  |  | HDL-C | ↑ |  |  |  |  |  |  |
|  |  |  |  |  |  |  |  |  |  |  |
|  |  | Patients with hyperlipidemia (67% with CAD) | LDL-C | ↓ | 6W | 207 | 61.5(18-80) |  |  |  |
|  |  |  | TG | ↓ |  |  |  |  |  |  |
|  |  |  | CHO | ↓ |  |  |  |  |  |  |
|  |  |  | HDL-C | ↑ |  |  |  |  |  |  |
|  |  |  |  |  |  |  |  |  |  |  |

^a^ ↓ means that the case group is significantly lower than the control group, or the post-control group is significantly lower than pre-control group; ↑ means that the case group is significantly higher than the control group, or the post-control group is significantly higher than pre-control group.

# Table S2 The literatures on the effect of β-blockers on the cardiovascular indicators

| ID | Drug names | Subjects | Indicators | Change ^a^ | Medication Duration | Sample Size  (n) | Age | References | Design | NOS/  CAMARADES  Score |
| --- | --- | --- | --- | --- | --- | --- | --- | --- | --- | --- |
| 1 | Metoprolol | Patients with CHF | IL-6 | ↓ | 6M | 48 | 61±12 | (37) | pre- and post-control | 8 |
|  |  |  | TNF-α | ↓ |  |  |  |  |  |  |
|  |  |  |  |  |  |  |  |  |  |  |
| 2 | Metoprolol/Bisoprolol | Patients with IDCM | TNF-α | ↓ | 12W | 32 | 61(26-74) | (38) | pre- and post-control | 8 |
|  |  |  |  |  |  |  |  |  |  |  |
| 3 | Propranolol/Atenolol/  Metoprolol/Carvedilol | Patients with unstable angina | CRP | ↓ | admitted to hospital | 20:30 | 60±11:60±10 | (39) | case-control | 8 |
|  |  |  |  | ↓ | 24H |  |  |  |  |  |
|  |  |  |  | ↓ | 48H |  |  |  |  |  |
|  |  |  |  | ↓ | out of the hospital |  |  |  |  |  |
|  |  |  |  |  |  |  |  |  |  |  |
|  |  |  | IL-6 | ↓ | admitted to hospital | 20:30 | 60±11:60±10 |  |  |  |
|  |  |  |  | ↓ | 24H |  |  |  |  |  |
|  |  |  |  |  |  |  |  |  |  |  |
| 4 | Nebivolol | Patients with hypertension | hsCRP | ↓ | 12W | 15 | —— | (40) | pre- and post-control | 7 |
|  | Atenolol | Patients with hypertension | hsCRP | ↓ | 12W | 15 | —— |  |  |  |
|  |  |  |  |  |  |  |  |  |  |  |
| 5 | Atenolol | Patients with high risk of CAD | ET-1 | ↓ | 4W | 22 | —— | (41) | pre- and post-control | 8 |
| ID | Drug names | Subjects | Indicators | Change ^a^ | Medication Duration | Sample Size  (n) | Age | References | Design | NOS/  CAMARADES  Score |
| 6 | Carvedilol | Patients with hypertension | ET-1 | ↓ | 3M | 35 | 66.3±4.7 | (42) | pre- and post-control | 8 |
|  |  |  |  |  |  |  |  |  |  |  |
| 7 | Atenolol | Patients with hypertension | FIB | ↓ | 6M | 26 | 40-65 | (43) | pre- and post-control | 8 |
|  |  |  |  |  |  |  |  |  |  |  |
| 8 | Celiprolol | Patients with hypertension | FIB | ↓ | 12M | 12 | 39.8 | (44) | pre- and post-control | 8 |
|  |  |  | CHO | ↓ |  |  |  |  |  |  |
|  |  |  | LDL-C | ↓ |  |  |  |  |  |  |
|  |  |  | TG | ↓ |  |  |  |  |  |  |
|  |  |  |  |  |  |  |  |  |  |  |
| 9 | Nebivolol | Patients with hypertension | CHO | ↓ | 6W | 6376 | >65 | (45) | pre- and post-control | 9 |
|  |  |  | TG | ↓ |  |  |  |  |  |  |
|  |  |  |  |  |  |  |  |  |  |  |
| 10 | Nebivolol | Patients with hypertension | CHO | ↓ | 12W | 26 | 51.5±10.4 | (46) | pre- and post-control | 8 |
|  |  |  | LDL-C | ↓ |  |  |  |  |  |  |

^a^ ↓ means that the case group is significantly lower than the control group, or the post-control group is significantly lower than pre-control group; ↑ means that the case group is significantly higher than the control group, or the post-control group is significantly higher than pre-control group.

# Table S3 The literatures on the effect of Ca-channel blockers on the cardiovascular indicators

| ID | Drug names | Subjects | Indicators | Change ^a^ | Medication Duration | Sample Size  (n) | Age | References | Design | NOS/  CAMARADES  Score |
| --- | --- | --- | --- | --- | --- | --- | --- | --- | --- | --- |
| 1 | Nifedipine | DBA/2 mice inoculated with encephalomyocarditis virus | IL-6 | ↓ | 5D | 10:10 | 4 W | (47) | case-control | 7 |
|  |  |  | TNF-α | ↓ |  |  |  |  |  |  |
|  |  |  | MMP-2 | ↓ |  |  |  |  |  |  |
|  |  |  | MMP-9 | ↓ |  |  |  |  |  |  |
|  |  |  |  |  |  |  |  |  |  |  |
| 2 | Barnidipine | Patients with hypertension | IL-6 | ↓ | 6M | 149 | —— | (48) | pre- and post-control | 8 |
|  |  |  | TNF-α | ↓ |  |  |  |  |  |  |
|  |  |  | hsCRP | ↓ |  |  |  |  |  |  |
|  |  |  |  |  |  |  |  |  |  |  |
| 3 | Nifedipine | Patients with hypertension | TNF-α | ↓ | 12W | 83 | 63.02±2.15 | (49) | pre- and post-control | 8 |
|  |  |  | CRP | ↓ |  |  |  |  |  |  |
|  |  |  |  |  |  |  |  |  |  |  |
| 4 | Lercanidipine | Patients with hypertension | MMP-9 | ↓ | 15D | 7 | 48.9±5.3 | (50) | pre- and post-control | 8 |
|  |  |  |  |  |  |  |  |  |  |  |
| 5 | Barnidipine | Patients with hypertension | hsCRP | ↓ | 6M | 75 | 60.5±8.9 | (51) | pre- and post-control | 8 |
|  |  |  | TNF-α | ↓ |  |  |  |  |  |  |
|  |  |  | MMP-2 | ↓ |  |  |  |  |  |  |
|  |  |  | MMP-9 | ↓ |  |  |  |  |  |  |
|  |  |  |  |  |  |  |  |  |  |  |
|  | Lercanidipine | Patients with hypertension | MMP-2 | ↓ | 6M | 76 | 60.7±8.8 |  |  |  |
|  |  |  | MMP-9 | ↓ |  |  |  |  |  |  |

^a^ ↓ means that the case group is significantly lower than the control group, or the post-control group is significantly lower than pre-control group; ↑means that the case group is significantly higher than the control group, or the post-control group is significantly higher than pre-control group.

# Table S4 The literatures on the effect of Clopidogrel on the cardiovascular indicators

| ID | Drug names | Subjects | Indicators | Change ^a^ | Medication Duration | Sample Size  (n) | Age | References | Design | NOS/  CAMARADES  Score |
| --- | --- | --- | --- | --- | --- | --- | --- | --- | --- | --- |
| 1 | Clopidogrel | Patients with ACS | TNF-α | ↓ | 30D | 57:58 | 53.5±14.9:52.5±16.9 | (52) | case-control | 8 |
|  |  |  | hsCRP | ↓ |  |  |  |  |  |  |
|  |  |  |  |  |  |  |  |  |  |  |
| 2 | Clopidogrel | Patients with SCAD | TNF-α | ↓ | 1Y | 101 | 61(52,79) | (53) | pre- and post-control | 9 |
|  |  |  |  |  |  |  |  |  |  |  |
| 3 | Clopidogrel | Patients with SCAD | hsCRP | ↓ | 5W | 77:26 | 64±4:65±3 | (54) | case-control | 9 |
|  |  |  | CD40L | ↓ |  |  |  |  |  |  |
|  |  |  |  |  |  |  |  |  |  |  |
| 4 | Clopidogrel | Patients with SCAD | CD40L | ↓ | 8W | 37:36 | 64±11:66±8 | (55) | case-control | 8 |
|  |  |  |  |  |  |  |  |  |  |  |
| 5 | Clopidogrel | Patients with CAD | CD62P | ↓ | ＞3M | 8:17 | 72(50-81):71(46-88) | (56) | case-control | 8 |
|  |  |  |  |  |  |  |  |  |  |  |
| 6 | Clopidogrel | Patients with ACS | sCD40L | ↓ | 24H | 23 | 58±10 | (57) | pre- and post-control | 8 |
|  |  |  | sCD62P | ↓ |  |  |  |  |  |  |
|  |  |  |  |  |  |  |  |  |  |  |
| 7 | Clopidogrel | Patients with unstable angina | MMP-2 | ↓ | 4W | 35 | 64.2±6.7 | (58) | case-control | 8 |
|  |  |  | MMP-9 | ↓ |  |  |  |  |  |  |
|  |  |  |  |  |  |  |  |  |  |  |
| 8 | Clopidogrel | Patients with ischemic stroke | MMP-9 | ↓ | 4W | 35:35 | 64.1±7.1:63.7±6.7 | (59) | case-control | 8 |
| ID | Drug names | Subjects | Indicators | Change ^a^ | Medication Duration | Sample Size  (n) | Age | References | Design | NOS/  CAMARADES  Score |
| 9 | Clopidogrel | Patients with ischemic stroke | MMP-9 | ↓ | 2W | 37 | 68.1±7.8 | (60) | pre- and post-control | 8 |
|  |  |  |  |  |  |  |  |  |  |  |
| 10 | Clopidogrel | Mice with AMI | TNF-α | ↓ | 8W | 10:10 | —— | (61) | case-control | 8 |
|  |  |  | MMP-2 | ↓ |  |  |  |  |  |  |
|  |  |  | MMP-9 | ↓ |  |  |  |  |  |  |

^a^ ↓ means that the case group is significantly lower than the control group, or the post-control group is significantly lower than pre-control group; ↑means that the case group is significantly higher than the control group, or the post-control group is significantly higher than pre-control group.

# Table S5 The literatures on the effect of Aspirin on the cardiovascular indicators

| ID | Drug names | Subjects | Indicators | Change ^a^ | Medication Duration | Sample Size  (n) | Age | References | Design | NOS/  CAMARADES  Score |
| --- | --- | --- | --- | --- | --- | --- | --- | --- | --- | --- |
| 1 | Aspirin | Patients with chronic stable angina | IL-6 | ↓ | 3W | 40 | 55±5 | (62) | pre- and post-control | 8 |
|  |  |  | CRP | ↓ |  |  |  |  |  |  |
|  |  |  |  |  |  |  |  |  |  |  |
| 2 | Aspirin | Patients with angina | IL-6 | ↓ | 4W | 30 | 47.32±5.54 | (63) | pre- and post-control | 8 |
|  |  |  | TNF-α | ↓ |  |  |  |  |  |  |
|  |  |  | hsCRP | ↓ |  |  |  |  |  |  |
|  |  |  |  |  |  |  |  |  |  |  |
| 3 | Aspirin | Patients with angina | TNF-α | ↓ | 4W | 40 | —— | (64) | pre- and post-control | 8 |
|  |  |  | hsCRP | ↓ |  |  |  |  |  |  |
|  |  |  | CHO | ↓ |  |  |  |  |  |  |
|  |  |  |  |  |  |  |  |  |  |  |
| 4 | Aspirin | Patients with hypertension | sCD62P | ↓ | 3M | 35 | 64±7 | (65) | pre- and post-control | 8 |
|  |  |  |  |  |  |  |  |  |  |  |
| 5 | Aspirin | Patients with CAD | CD62P | ↓ | 2W | 41 | —— | (66) | pre- and post-control | 8 |
|  |  |  |  |  |  |  |  |  |  |  |
| 6 | Aspirin | Patients with angina | NO | ↑ | —— | 30 | 58.4±8.2 | (67) | case-control | 8 |
|  |  |  |  |  |  |  |  |  |  |  |
| 7 | Aspirin | Patients with SCAD | NO | ↑ | 12W | 37 | 64 | (68) | pre- and post-control | 8 |
| ID | Drug names | Subjects | Indicators | Change ^a^ | Medication Duration | Sample Size  (n) | Age | References | Design | NOS/  CAMARADES  Score |
| 8 | Aspirin | Patients with unstable angina | CHO | ↓ | 4W | 46 | 53.03±8.67 | (69) | pre- and post-control | 8 |
|  |  |  | LDL-C | ↓ |  |  |  |  |  |  |
|  |  |  |  |  |  |  |  |  |  |  |
| 9 | Aspirin | New Zealand rabbits with hyperlipidemia atherosclerotic | CHO | ↓ | 4W | —— | —— | (70) | pre- and post-control | 7 |
|  |  |  | LDL-C | ↓ |  | —— | —— |  |  |  |

^a^ ↓ means that the case group is significantly lower than the control group, or the post-control group is significantly lower than pre-control group; ↑means that the case group is significantly higher than the control group, or the post-control group is significantly higher than pre-control group.

# Table S6 The literatures on the effect of ACEI/ARB on the cardiovascular indicators

| ID | Drug names | Subjects | Indicators | Change ^a^ | Medication Duration | Sample Size  (n) | Age | References | Design | NOS/  CAMARADES  Score |
| --- | --- | --- | --- | --- | --- | --- | --- | --- | --- | --- |
| 1 | Irbesartan | Patients with CAD complicated with arterial hypertension | IL-6 | ↓ | 3M | 21 | 56±8 | (71) | pre- and post-control | 8 |
|  |  |  | hsCRP | ↓ |  |  |  |  |  |  |
|  |  |  | MMP-9 | ↓ |  |  |  |  |  |  |
|  |  |  |  |  |  |  |  |  |  |  |
|  | Enalapril | Patients with CAD complicated with arterial hypertension | MMP-9 | ↓ | 3M | 21 | 56±8 |  |  |  |
|  |  |  |  |  |  |  |  |  |  |  |
| 2 | Candesartan | Patients with congestive heart failure | IL-6 | ↓ | 14W | 44 | 55.6±3.3 | (72) | pre- and post-control | 8 |
|  |  |  | TNF-α | ↓ |  |  |  |  |  |  |
|  |  |  | sICAM-1 | ↓ |  |  |  |  |  |  |
|  |  |  |  |  |  |  |  |  |  |  |
| 3 | Losartan | Patients with hypertension | TNF-α | ↓ | 3M | 16 | 42±4 | (73) | pre- and post-control | 8 |
|  |  |  | TNF-α | ↓ | 6M | 16 | 42±4 |  |  |  |
|  |  |  |  |  |  |  |  |  |  |  |
| 4 | Ramipril | Patients with high cardiovascular risk | hsCRP | ↓ | 6M | 77 | 60±11 | (74) | pre- and post-control | 8 |
|  |  |  |  |  |  |  |  |  |  |  |
| 5 | Ramipril | SD rats with hypercholesterolemia | ICAM-1 | ↓ | 10W | 10:10 | —— | (75) | pre- and post-control | 7 |
|  | Losartan | SD rats with hypercholesterolemia | ICAM-1 | ↓ | 10W | 10:10 | —— |  |  |  |
|  |  |  |  |  |  |  |  |  |  |  |
| ID | Drug names | Subjects | Indicators | Change ^a^ | Medication Duration | Sample Size  (n) | Age | References | Design | NOS/  CAMARADES  Score |
| 6 | Irbesartan | Patients with hypertension | FIB | ↓ | 6M | 28 | 40-65 | (43) | pre- and post-control | 8 |
|  |  |  |  |  |  |  |  |  |  |  |
| 7 | Perindopril | Patients with hypertension | FIB | ↓ | 6W | 28 | 43-64 | (76) | pre- and post-control | 8 |
|  |  |  |  |  |  |  |  |  |  |  |
| 8 | Candesartan | Patients with hypertension | MMP-9 | ↓ | 3M | 17 | 47±8 | (77) | pre- and post-control | 8 |
|  | Lisinopril | Patients with hypertension | MMP-9 | ↓ | 3M | 16 | 47±8 |  |  |  |

^a^ ↓ means that the case group is significantly lower than the control group, or the post-control group is significantly lower than pre-control group; ↑means that the case group is significantly higher than the control group, or the post-control group is significantly higher than pre-control group.

# Table S7 Analysis model of each indicator and the interpretation contribution rate of its variables to the model

|  | Models | %change of IQR | 95%CI | p-value | AIC | Variables | R^2^ | Contribution |
| --- | --- | --- | --- | --- | --- | --- | --- | --- |
| IL-6 | $\log\left( Indicator \right)\sim{PM}_{2.5}+Age+Gender+BMI+{ED}^{a}+\left( 1 \vert ID \right)$ | 1.74 | (-20.72,29.82) | 0.891 | 135.75 | Model | 0.110 |  |
|  |  |  |  |  |  | ED | 0.074 | 67.27% |
|  |  |  |  |  |  | Age | 0.052 | 47.27% |
|  |  |  |  |  |  | BMI | 0.009 | 8.18% |
|  |  |  |  |  |  | Gender | 0.001 | 0.91% |
|  |  |  |  |  |  | PM_2.5_ | <0.001 | <0.01% |
|  |  |  |  |  |  |  |  |  |
| TNF-α | $\log\left( Indicator \right)\sim{PM}_{2.5}+Age+Gender+BMI+ED+\left( 1 \vert ID \right)$ | -2.57 | (-7.33,2.31) | 0.293 | 51.36 | Model | 0.098 |  |
|  |  |  |  |  |  | Gender | 0.057 | 58.16% |
|  |  |  |  |  |  | Age | 0.029 | 29.59% |
|  |  |  |  |  |  | ED | 0.008 | 8.16% |
|  |  |  |  |  |  | PM_2.5_ | 0.002 | 2.04% |
|  |  |  |  |  |  | BMI | <0.001 | <0.01% |
|  |  |  |  |  |  |  |  |  |
| CRP | $\log\left( Indicator \right)\sim{PM}_{2.5}+Gender+BMI+{Temp}^{b}+ED+\left( 1 \vert ID \right)$ | -23.43 | (-39.03, -3.62) | 0.027 | 127.5 | Model | 0.143 |  |
|  |  |  |  |  |  | PM_2.5_ | 0.078 | 54.55% |
|  |  |  |  |  |  | ED | 0.076 | 53.15% |
|  |  |  |  |  |  | Temp | 0.018 | 12.59% |
|  |  |  |  |  |  | BMI | 0.015 | 10.49% |
|  |  |  |  |  |  | Gender | <0.001 | <0.01% |
|  | Models | %change of IQR | 95%CI | p-value | AIC | Variables | R^2^ | Contribution |
| FIB | $\log\left( Indicator \right)\sim{PM}_{2.5}+Age+Gender+BMI+ED+\left( 1 \vert ID \right)$ | -2.81 | (-8.90,2.94) | 0.37 | 30.71 | Model | 0.234 |  |
|  |  |  |  |  |  | Age | 0.142 | 60.68% |
|  |  |  |  |  |  | BMI | 0.078 | 33.33% |
|  |  |  |  |  |  | Gender | 0.073 | 31.20% |
|  |  |  |  |  |  | ED | 0.036 | 15.38% |
|  |  |  |  |  |  | PM_2.5_ | 0.016 | 6.84% |
|  |  |  |  |  |  |  |  |  |
| CD62P | $\log\left( Indicator \right)\sim{PM}_{2.5}+Gender+BMI+Temp+ED+\left( 1 \vert ID \right)$ | -1.14 | (-5.61,4.42) | 0.649 | 23.5 | Model | 0.194 |  |
|  |  |  |  |  |  | BMI | 0.153 | 78.87% |
|  |  |  |  |  |  | ED | 0.044 | 22.68% |
|  |  |  |  |  |  | PM_2.5_ | 0.002 | 1.03% |
|  |  |  |  |  |  | Temp | <0.001 | <0.01% |
|  |  |  |  |  |  | Gender | <0.001 | <0.01% |
|  |  |  |  |  |  |  |  |  |
| CD40L | $\log\left( Indicator \right)\sim{PM}_{2.5}+Gender+BMI+Temp+ED+\left( 1 \vert ID \right)$ | 1.76 | (-7.24,16.36) | 0.728 | 65.41 | Model | 0.060 |  |
|  |  |  |  |  |  | Temp | 0.044 | 73.33% |
|  |  |  |  |  |  | PM_2.5_ | 0.002 | 3.33% |
|  |  |  |  |  |  | ED | 0.001 | 1.67% |
|  |  |  |  |  |  | Gender | <0.001 | <0.01% |
|  |  |  |  |  |  | BMI | <0.001 | <0.01% |
|  | Models | %change of IQR | 95%CI | p-value | AIC | Variables | R^2^ | Contribution |
| ICAM-1 | $\log\left( Indicator \right)\sim{PM}_{2.5}+Age+Gender+BMI+ED+\left( 1 \vert ID \right)$ | -1.18 | (-3.60,1.28) | 0.335 | 27.73 | Model | 0.204 |  |
|  |  |  |  |  |  | BMI | 0.184 | 90.20% |
|  |  |  |  |  |  | ED | 0.038 | 18.63% |
|  |  |  |  |  |  | Age | 0.027 | 13.24% |
|  |  |  |  |  |  | Gender | 0.021 | 10.29% |
|  |  |  |  |  |  | PM_2.5_ | <0.001 | <0.01% |
|  |  |  |  |  |  |  |  |  |
| NO | $\log\left( Indicator \right)\sim{PM}_{2.5}+Gender+BMI+Temp+ED+\left( 1 \vert ID \right)$ | -10.31 | (-25.49,10.42) | 0.284 | 115.94 | Model | 0.153 |  |
|  |  |  |  |  |  | Gender | 0.066 | 43.14% |
|  |  |  |  |  |  | Temp | 0.042 | 27.45% |
|  |  |  |  |  |  | BMI | 0.031 | 20.26% |
|  |  |  |  |  |  | PM_2.5_ | 0.022 | 14.38% |
|  |  |  |  |  |  | ED | 0.008 | 5.23% |
|  |  |  |  |  |  |  |  |  |
| ET-1 | $\log\left( Indicator \right)\sim{PM}_{2.5}+Age+Gender+BMI+ED+\left( 1 \vert ID \right)$ | -6.2 | (-12.98,0.82) | 0.082 | 81.94 | Model | 0.144 |  |
|  |  |  |  |  |  | Gender | 0.096 | 66.67% |
|  |  |  |  |  |  | Age | 0.068 | 47.22% |
|  |  |  |  |  |  | ED | 0.021 | 14.58% |
|  |  |  |  |  |  | BMI | 0.013 | 9.03% |
|  |  |  |  |  |  | PM_2.5_ | 0.005 | 3.47% |
|  | Models | %change of IQR | 95%CI | p-value | AIC | Variables | R^2^ | Contribution |
| MMP-2 | $\log\left( Indicator \right)\sim{PM}_{2.5}+Age+Gender+BMI+ED+\left( 1 \vert ID \right)$ | 3.9 | (-0.91,8.44) | 0.097 | 11.58 | Model | 0.058 |  |
|  |  |  |  |  |  | PM_2.5_ | 0.035 | 60.34% |
|  |  |  |  |  |  | Age | 0.021 | 36.21% |
|  |  |  |  |  |  | BMI | 0.007 | 12.07% |
|  |  |  |  |  |  | ED | 0.002 | 3.45% |
|  |  |  |  |  |  | Gender | <0.001 | <0.01% |
|  |  |  |  |  |  |  |  |  |
| MMP-9 | $\log\left( Indicator \right)\sim{PM}_{2.5}+Gender+Temp+{RH}^{c}+ED+\left( 1 \vert ID \right)$ | -4.59 | (-14.90,7.48) | 0.448 | 83.04 | Model | 0.261 |  |
|  |  |  |  |  |  | Gender | 0.153 | 58.62% |
|  |  |  |  |  |  | RH | 0.148 | 56.70% |
|  |  |  |  |  |  | Temp | 0.075 | 28.74% |
|  |  |  |  |  |  | ED | 0.037 | 14.18% |
|  |  |  |  |  |  | PM_2.5_ | 0.012 | 4.60% |
|  |  |  |  |  |  |  |  |  |
| CHO | $\log\left( Indicator \right)\sim{PM}_{2.5}+Age+Gender+BMI+ED+\left( 1 \vert ID \right)$ | 3.66 | (-0.75,8.85) | 0.129 | 12.74 | Model | 0.494 |  |
|  |  |  |  |  |  | Gender | 0.342 | 69.23% |
|  |  |  |  |  |  | ED | 0.126 | 25.51% |
|  |  |  |  |  |  | BMI | 0.105 | 21.26% |
|  |  |  |  |  |  | PM_2.5_ | 0.033 | 6.68% |
|  |  |  |  |  |  | Age | 0.001 | 0.20% |
|  | Models | %change of IQR | 95%CI | p-value | AIC | Variables | R^2^ | Contribution |
| TG | $\log\left( Indicator \right)\sim{PM}_{2.5}+Age+Gender+BMI+ED+\left( 1 \vert ID \right)$ | -2.34 | (-9.96,6.71) | 0.583 | 67.26 | Model | 0.336 |  |
|  |  |  |  |  |  | ED | 0.196 | 58.33% |
|  |  |  |  |  |  | BMI | 0.176 | 52.38% |
|  |  |  |  |  |  | Gender | 0.162 | 48.21% |
|  |  |  |  |  |  | Age | 0.004 | 1.19% |
|  |  |  |  |  |  | PM_2.5_ | 0.002 | 0.60% |
|  |  |  |  |  |  |  |  |  |
| HDL-C | $\log\left( Indicator \right)\sim{PM}_{2.5}+Age+Gender+BMI+ED+\left( 1 \vert ID \right)$ | 4.09 | (-0.08,8.39) | 0.05 | 21.68 | Model | 0.144 |  |
|  |  |  |  |  |  | Gender | 0.063 | 43.75% |
|  |  |  |  |  |  | Age | 0.026 | 18.06% |
|  |  |  |  |  |  | PM_2.5_ | 0.013 | 9.03% |
|  |  |  |  |  |  | ED | 0.003 | 2.08% |
|  |  |  |  |  |  | BMI | <0.001 | <0.01% |
|  |  |  |  |  |  |  |  |  |
| LDL-C | $\log\left( Indicator \right)\sim{PM}_{2.5}+Age+Gender+BMI+ED+\left( 1 \vert ID \right)$ | 7.21 | (0.09,17.92) | 0.075 | 47.03 | Model | 0.314 |  |
|  |  |  |  |  |  | Gender | 0.215 | 68.47% |
|  |  |  |  |  |  | BMI | 0.083 | 26.43% |
|  |  |  |  |  |  | PM_2.5_ | 0.059 | 18.79% |
|  |  |  |  |  |  | ED | 0.016 | 5.10% |
|  |  |  |  |  |  | Age | <0.001 | <0.01% |
|  | Models | *β* ^d^ | 95%CI | p-value | AIC | Variables | R^2^ | Contribution |
| AIP | $\log\left( Indicator \right)\sim{PM}_{2.5}+Age+Gender+BMI+ED+\left( 1 \vert ID \right)$ | -0.001 | (-0.003,0.001) | 0.226 | 23.35 | Model | 0.340 |  |
|  |  |  |  |  |  | ED | 0.253 | 74.41% |
|  |  |  |  |  |  | BMI | 0.140 | 41.18% |
|  |  |  |  |  |  | Gender | 0.054 | 15.88% |
|  |  |  |  |  |  | Age | 0.012 | 3.53% |
|  |  |  |  |  |  | PM_2.5_ | 0.010 | 2.94% |

^a^ ED: effective drug

^b^ Temp: Temperature

^c^ RH: relative humidity;

^d^ AIP is normally distributed, so there is no need for logarithmic conversion. Therefore, there result shows the correlation coefficient β of PM_2.5_ instead of percentage change associated with a PM_2.5_ interquartile range decrease.

.
